# FAM65A as a novel prognostic biomarker in human tumors reveal by a pan-cancer analysis

**DOI:** 10.1007/s12672-021-00456-z

**Published:** 2021-12-07

**Authors:** Wenken Liang, Chune Mo, Jianfen Wei, Wei Chen, Weiwei Gong, Jianling Shi, Xianliang Hou, Chunhong Li, Yecheng Deng, Minglin Ou

**Affiliations:** 1 College of Life Science, Guangxi Normal University, No. 1, Yanshan Middle Road, Guilin, 541000 China; 2 Central Laboratory, Guangxi Health Commission Key Laboratory of Glucose and Lipid Metabolism Disorders, The Second Affiliated Hospital of Guilin Medical University, No. 212, Renmin Road, Guilin, 541000 China; 3 Gastrointestinal Surgery, The Second Affiliated Hospital of Guilin Medical University, Guilin, 541000 China

**Keywords:** FAM65A, RIPOR1, Prognostic biomarker, Pan-cancer, TCGA, GEO

## Abstract

**Background:**

Family with sequence similarity 65 member A (FAM65A), also known as RIPOR1, is differentially expressed between human tumor and non-tumor tissues in kinds of cancers. In addition, it was reported that the product of *FAM65A* may be a biomarker for cholangiocarcinoma patients. However, there is still no evidence on the relationship between the *FAM65A* and different types of tumors. Our study is mainly for exploring the prognostic values of *FAM65A* in pan-cancer and for further discovering a potential therapeutics target.

**Methods:**

We analyzed *FAM65A* expression, prognostic values, genetic alteration, protein phosphorylation, immune infiltration and enrichment analysis across different types of human malignant tumors based on The Cancer Genome Atlas (TCGA) and Gene Expression Omnibus (GEO) datasets. Additionally, Real-Time PCR (RT-qPCR) was performed to further confirm the roles of *FAM65A* in the pathogenesis of colorectal cancer.

**Results:**

We found that *FAM65A* expression was associated with the prognosis of multiple human tumors, especially colorectal cancer. Moreover, we also observed that *FAM65A* was highly expressed in colorectal cancer through RT-qPCR. We observed that decreasing phosphorylation level of the S351 locus in colon adenocarcinoma, uterine corpus endometrial carcinoma and lung adenocarcinoma. And the expression of *FAM65A* was positively related to cancer-associated fibroblasts (CAFs) infiltration in many tumors, such as colon adenocarcinoma. Therefore, *FAM65A* may be a potential prognostic biomarker of human tumors.

**Supplementary Information:**

The online version contains supplementary material available at 10.1007/s12672-021-00456-z.

## Background

There will be an estimated 18.1 million new cancer cases and 9.6 million cancer deaths in 2018, according to Global Cancer Statistics 2018 [1]. Tumors have gradually become the leading cause of death in the world [1]. With the continuous development of diagnosis and treatment technology, the survival time of cancer patients has been further improved. However, due to the complexity and stealthiness of the form and development of tumors, most patients don’t receive timely treatment, and resulting in poor prognosis. Therefore, it is necessary that novel markers of early diagnosis are needed to apply in medical services. Pan-cancer analysis is valuable to reveal the correlation between any gene of interest and its clinical prognosis as well as its potential molecular mechanisms across different tumors [2]. In addition, pan-cancer analysis can also span the breadth of analyses and identify commonalities, differences, and emerging themes in human cancers [3]. TCGA Pan-Cancer analysis project, which is funded by USA National Institutes of health, includes functional genomics data in different tumors and provides the possibility for us to perform pan-cancer analysis [4–6].

It is now clear that RHO proteins are involved in almost every stage of tumorigenesis and some of the studies indicated that reduced RHO-protein function contributes to the morphological changes observed in tumor cells; this raises the dangerous possibility that inhibition of RHO proteins might promote a more aggressive tumor phenotype [7]. RHO family interacting cell polarization regulator 1 (RIPOR1) is also known as FAM65A, a novel Rho effector protein, which associates with GTP-bound RHO proteins (RHOA, RHOB, and RHOC) via an N-terminal HR1 domain [8]. It is noteworthy that FAM65A plays important roles in RHO-regulated the polarity of migrating cells via Golgi reorientation [8]. In addition, it is reported that FAM65A protein may be a biomarker for cholangiocarcinoma patients [9]. Therefore, we think that *FAM65A* may be one of the important genes for tumorigenesis and related to the prognosis of cancer patients. However, there is still no evidence on the association between *FAM65A* and different types of tumors based on the big clinical data.

In our study, we have comprehensively analyzed the correlation between the *FAM65A* expression and prognostic value as well as immune infiltration in different types of tumors. In addition, we also have conducted a series of analysis of *FAM65A*, including mutation, protein phosphorylation and gene enrichment, and so on.

## Materials and methods

### Gene expression analysis

The tumor immune estimation resource, TIMER2.0 web server (http://timer.cistrome.org/ accessed 25 July 2021) was used to explore *FAM65A* expression difference between primary tumor and the control based on the TCGA database [10]. For certain tumors without the expression information of normal tissue in the TCGA database, whose expression profile data was obtained through the GEPIA2 (http://gepia2.cancer-pku.cn/#index accessed 25 July 2021) based on the GTEx database, under the setting of |Log_2_FC| Cutoff = 1, P-value cutoff = 0.01 and “Match TCGA normal and GTEx data” [11]. To further confirm the expression difference of *FAM65A* between primary tumor and the control, the Oncomine web server (https://www.oncomine.org accessed 1 August 2021) was used to obtain the expression profile data.

In addition, we also tried to explore the association between *FAM65A* expression and the pathological stages of tumor through the GEPIA2 web server (accessed 25 July 2021). The violin plots indicate *FAM65A* expression in main stages (stage I, II, III, and IV) of different types of tumors.

UALCAN (http://ualcan.path.uab.edu/analysis-prot.html accessed 25 July 2021), an interactive web-portal for in-depth analysis of TCGA gene expression data, provides the possibility for us to conduct protein expression analysis of the CPTAC dataset [12, 13].

### The prognostic analysis of *FAM65A*

The GEPIA2 web server (accessed 28 July 2021) was used to explore the *FAM65A* prognostic values in different types of tumors [11]. The significant survival maps of overall survival (OS) and disease-free survival (DFS) were obtained through the GEPIA2 web server, under setting of cutoff-high (50%) and cutoff-low (50%) values. Additionally, the Oncolnc web server (http://www.oncolnc.org/ accessed 16 August 2021) was also used to confirm the *FAM65A* prognostic values in colorectal cancer. Finally, the GSE17536 (probe name: 218029_at) cohort was downloaded to conduct statistical analysis from the PrognoScan (http://dna00.bio.kyutech.ac.jp/PrognoScan/index.html accessed 15 September 2021), in order to further confirm *FAM65A* in prognostic values of colon adenocarcinoma. We used the SPSS Statistics 23.0 to perform univariate and multivariable Cox analysis.

### Real-time PCR

In this study, we have collected a few of human tissues from the Second Affiliated Hospital of Guilin Medical College in China, including colorectal cancer (n = 6) and non-tumor tissues (n = 4). All patients with colorectal cancer have signed an informed consent form. In addition, the ethics committee of the Second Affiliated Hospital of Guilin Medical College has approved the study. Total mRNA of samples was extracted using TRIzol (Invitrogen life technologies). Total mRNA was reverse transcribed to generate cDNA using the RT cDNA Synthesis kit. Quantitative polymerase chain reaction (qPCR) was performed using ViiA 7 Real-time PCR System (Applied Biosystems) and three replicates were performed for each sample.

The primer sequences were used:

- FAM65A
- Forward: 5′-GGCGAGTTTCATCTCCGAAT-3′,
- Reverse: 5′-AGACACTGCCCACAACCACA-3′,
- β-Actin
- Forward: 5′-GTGGCCGAGGACTTTGATTG-3′,
- Reverse: 5′-CCTGTAACAACGCATCTCATATT-3′.

2ΔΔCT method was used to quantify the relative *ZFC3H1* mRNA expression [14]. SPSS was used to perform statistical analyses (T test), and P < 0.05 was deemed statistically significant.

### *FAM65A* mutation in different types of tumors

The cBioPortal web server (http://www.cbioportal.org accessed 1 August 2021) is a comprehensive website, which explore, visualize and analyze multidimensional cancer genomics data [15]. In this study, the genetic alteration feature of *FAM65A* was obtained through the “Cancer Types Summary” module of the cBioPortal web server (The gene name recognized by the system is RIPOR1). In addition, the correlation between *FAM65A* genetic alteration and survival prognosis (DFS, DSS, OS and PFS) was explored in the “Comparison” module. The prognostic values are presented with log-rank P values.

### Immune infiltration analysis

We used the TIMER2.0 web server (accessed 18 August 2021) to evaluate the association between *FAM65A* expression and immune infiltration across different types of tumors. The result was analyzed through the Spearman’s correlation test based on EPIC, MCPCOUNTED, XCELL and TIDE algorithms.

### Gene enrichment analysis

The top 100 genes that are correlated with *FAM65A* were screened through “Similar Gene Detection” module of the GEPIA2 (accessed 22 August 2021). And the Pearson correlation between *FAM65A* and the top 5 genes is presented at the heatmap through the TIMER2.0 web server (accessed 26 August 2021). Furthermore, the Multi Experiment Matrix (https://biit.cs.ut.ee/mem/index.cgi accessed 21 August 2021) was used to screen the top 50 genes that are co-expressed with *FAM65A*.

The Jvenn (http://bioinformatics.psb.ugent.be/webtools/Venn/ accessed 21 August 2021), an interactive Venn diagram viewer [16], which was used to compare an intersection between the co-expressed genes and the correlated genes. Moreover, two sets of genes that were combined to conduct KEGG pathway analysis through the DAVID tool (https://david.ncifcrf.gov/ accessed 21 August 2021) and the “ggplot2” R packages.

At the same time, we used the Metascape web server (http://metascape.org/ accessed 26 August 2021) to perform GO enrichment analysis. After uploading the combined genes, “H sapiens” was selected to perform custom analysis, with the settings of Min Overlap = 3, P-value Cutoff = 0.01 and Min Enrichment = 1.5. Finally, the STRING web server (https://string-db.org/ accessed 26 August 2021) was used to screen the bound proteins with *FAM65A*. Entering the “FAM65A” for protein name, we selected “Homo sapiens” to obtain the PPI networks of FAM65A protein, with the main settings of network type (“full STRING network”), network edges (“evidence”), active interaction sources (“Experiments”), minimum required interaction score [“low confidence (0.150)”], max number of interactors to show (1st shell: “no more than 50 interactors”) and network display mode (“interactive svg”).

## Results

### Gene expression analysis

**Fig. 1:**
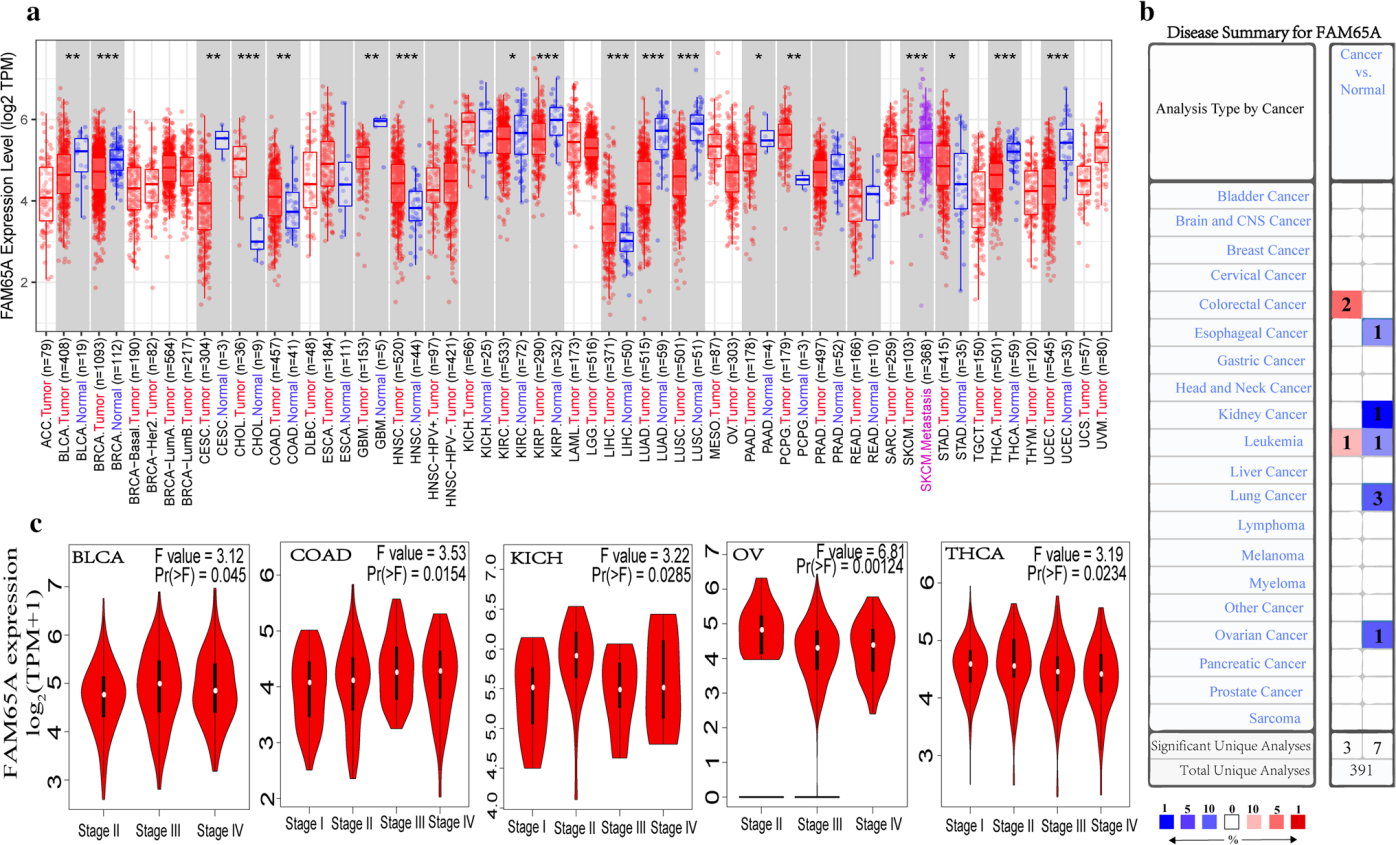
The expression level of FAM65A gene in different tumors. **a** The expression levels of FAM65A in human tumors and corresponding normal tissues are obtained through the TIMER2. **b** The expression levels of FAM65A in human tumors and corresponding normal tissues are obtained through the oncomine. **c** The FAM65A expression violin plots in different pathological stages of BLCA, COAD, KICH, OV and THCA are obtained through the GEPIA2. *P < 0.05, **P < 0.01, ***P < 0.001

Firstly, the TIMER2.0 was used to evaluate *FAM65A* expression difference between primary tumor and the control based on TCGA database. We found that *FAM65A* expression levels in tumor tissue of cholangio carcinoma (CHOL), head and neck squamous cell carcinoma (HNSC), hepatocellular carcinoma (LIHC), colon adenocarcinoma (COAD), pheochromocytoma and paraganglioma (PCPG) and stomach adenocarcinoma (STAD) are higher than the corresponding normal tissue based on TCGA dataset (Fig. 1a). In contrary, *FAM65A* expression levels in tumor tissue of breast invasive carcinoma (BRCA), kidney renal papillary cell carcinoma (KIRP), lung adenocarcinoma (LUAD), lung squamous cell carcinoma (LUSC), uterine corpus endometrial carcinoma (UCEC), cervical squamous cell carcinoma and endocervical adenocarcinoma (CESC), Glioblastoma multiforme (GBM), kidney renal clear cell carcinoma (KIRC) and Pancreatic adenocarcinoma (PAAD) are lower than the corresponding normal tissue based on TCGA dataset (Fig. 1a). Moreover, the normal tissues in the GTEx dataset serve as the controls and the results are presented in Fig. S1.

At the same time, the Oncomine web server was used to further confirm the expression difference between primary tumor and the control. The suppressed expression level of *FAM65A* was observed in esophageal cancer, kidney cancer, lung cancer and ovarian cancer, while elevated expression level of *FAM65A* was observed in colorectal cancer, compared with the control (Fig. 1b). In addition, the UALCAN tool was used to evaluate the FAM65A total protein in different types of tumors based on the CPTAC dataset. And the results are presented in Fig. S1. Finally, we used the GEPIA2 to observe the association between *FAM65A* expression and different pathological stages of tumor, such as colon adenocarcinoma (Fig. 1c).

### The prognostic analysis of *FAM65A*

**Fig. 2:**
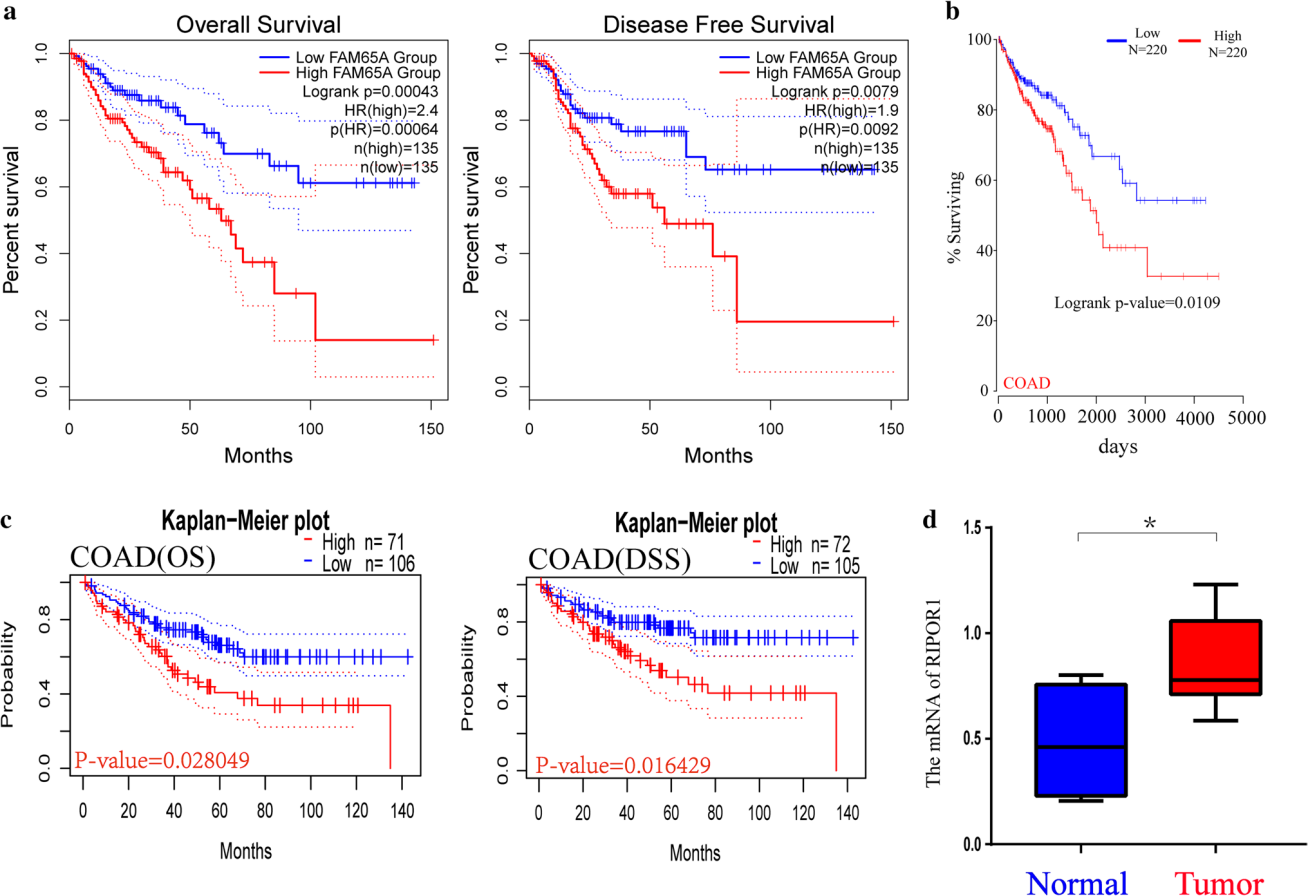
Relationship between FAM65A gene expression and the prognostic values in colorectal cancer. **a** The FAM65A prognostic values were obtained through the GEPIA2 tool. **b** The FAM65A prognostic value was obtained through the Oncolnc. **c** The FAM65A prognostic values were obtained based on the GSE17536 cohort. **d** RT-qPCR

We used the GEPIA tool to investigate the correlation between *FAM65A* expression and the survival prognosis of patients across different types of tumors. And the relationship between *FAM65A* expression and the prognostic values of pan-cancer is presented in Fig. S2. In this study, we focused on colorectal cancer and the highly expressed *FAM65A* was linked to poor overall survival and disease-free survival in the COAD patients (Fig. 2a). At the same time, the Oncolnc web server was used to further confirm the prognostic value of COAD and the result is presented in Fig. 2b.

In addition, the GSE17536 (probe name: 218029_at) cohort was downloaded to perform statistical analysis. COAD patients with high *FAM65A* expression had shorter overall survival and disease-specific survival in the GSE17536 cohort (Fig. 2c). Additionally, cox univariable analysis indicated clinical stage (HR = 3.623, P < 0.001), cell differentiation (HR = 2.133, P = 0.006) and *FAM65A* expression (HR = 3.789, P = 0.010) influenced the prognosis of COAD patients. However, multivariable cox analysis suggested *FAM65A* expression (HR = 4.400, P = 0.007) wasn’t an independent factor to influence COAD prognosis (Table 1).

**Table 1:** Univariate and multivariate cox analysis of clinical characteristics for COAD patients in GSE17536 cohort

Clinical variables	GSE17536 (probe name:213065_at)				GSE17536 (probe name:213065_at)			
	Univariate analysis				Multivariate analysis			
	HR	HR.95L	HR.95H	P value	HR	HR.95L	HR.95H	P value
Age	0.997	0.977	1.017	0.746	1.010	0.986	1.035	0.407
Gender	0.843	0.491	1.446	0.534	0.994	0.556	1.777	0.984
Differentiation	2.133	1.241	3.665	**0.006**	1.569	0.864	2.850	0.139
Clinical stage	3.623	2.495	5.262	**0.000**	3.923	2.607	5.903	**0.000**
FAM65A	3.789	1.376	10.431	**0.010**	4.400	1.494	12.960	**0.007**

Bold values indicate statistically significant factors that affect the prognosis of COAD patients

*Differentiation* Differentiation characteristics of cancer cells, *HR* hazard ratio

Finally, RT-qPCR were conducted to explore the expression difference between tumor and non-tumor tissues of colorectal cancer. As shown in Fig. 2d, we observed that the *FAM65A* relative expression level of tumors were higher than non-tumor tissues in colorectal cancer (P < 0.05), which was similar to the result observed by the bioinformatics analysis.

### *FAM65A* mutation in different types of tumors

**Fig. 3:**
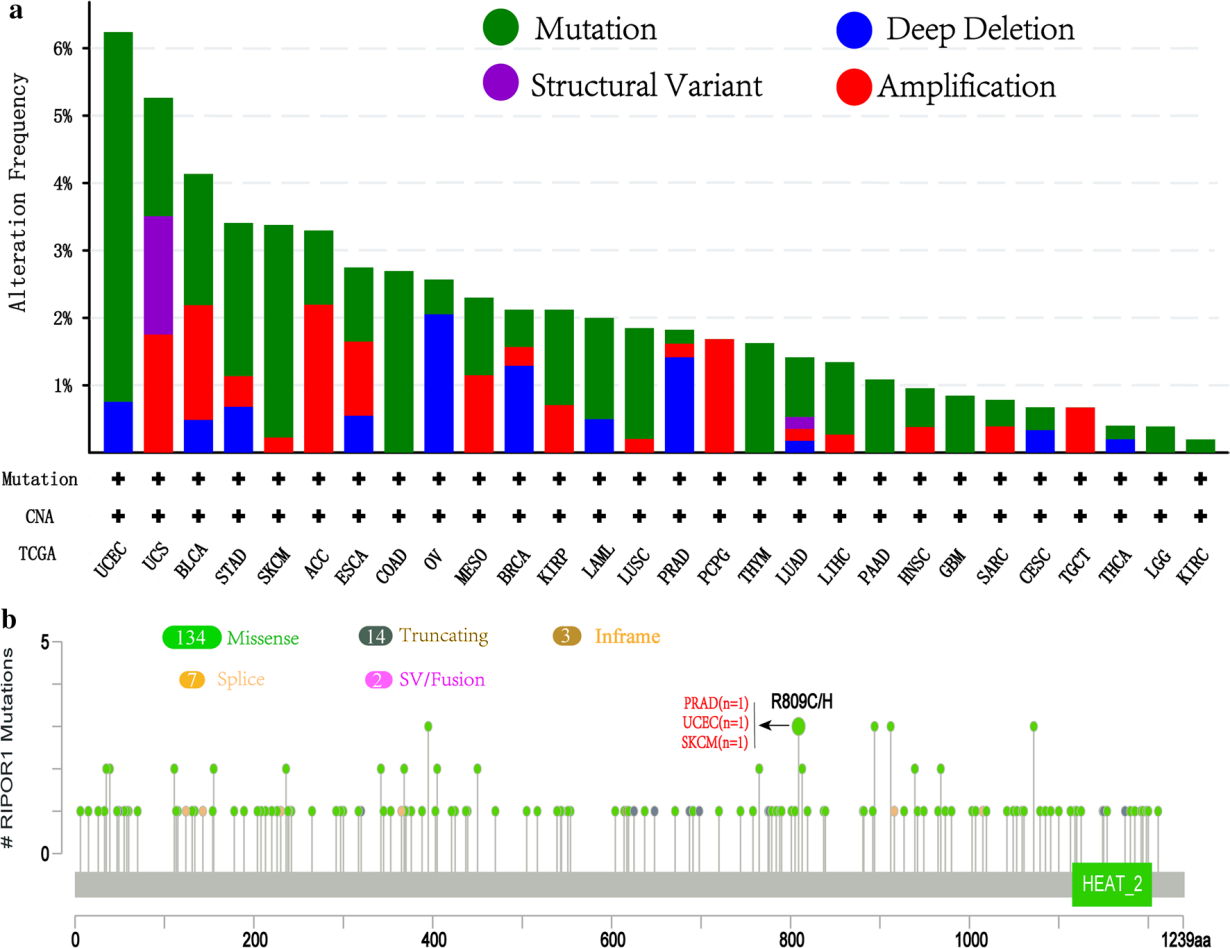
Mutation feature of FAM65A in different tumors of TCGA dataset. The alteration frequency with mutation type (**a**) and mutation site (**b**) are showed

The genetic alteration feature of *FAM65A* in kinds of tumors was explored based on the TCGA cohorts. As shown in Fig. 3a, we found that the patients with UCEC had the highest alteration frequency of *FAM65A* (> 6%) with “mutation” as the main type. “Mutation” as the unique form of genetic alteration existed in all patients with COAD, thymoma (THYM), PAAD, GBM, brain lower grade glioma (LGG) and KIRC. The sites, types and case number of *FAM65A* genetic alteration are presented in Fig. 3b.

We observed that the primary type of *FAM65A* genetic alteration was missense mutation. R809C/H alteration, which was found in one case of PRAD, one case of UCEC and one case of SKCM, leads to a frame shift mutation of *FAM65A*, translation from R (Arginine) to C (Cysteine) or H (Histidine) at the 809 site of FAM65A protein and FAM65A protein was subsequently truncated. In addition, the potential correlation between *FAM65A* genetic alteration and the prognosis with UCEC, prostate adenocarcinoma (PRAD) and skin cutaneous melanoma (SKCM) is presented in Fig. S3.

### FAM65A protein phosphorylation

**Fig. 4:**
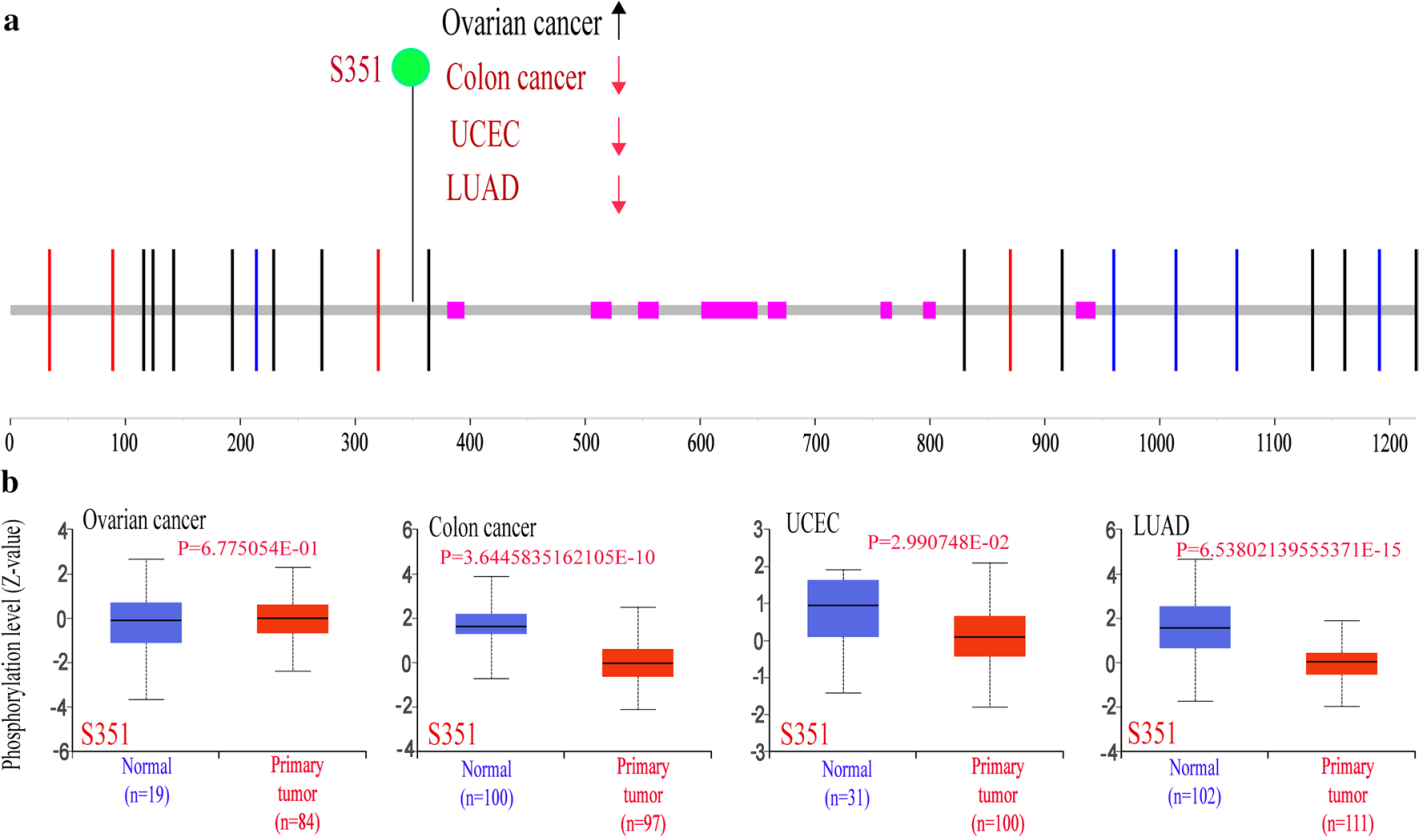
Phosphorylation analysis of RIPOR1 protein in different tumors. **a** The phosphoprotein site (S351) is showed in the schematic diagram of the RIPOR1 protein. **b** The box plots of different tumors, including ovarian cancer, colon cancer, UCEC and LUAD

The difference of FAM65A phosphorylation level in different tumors was explored based on the CPTAC dataset. Figure 4 indicates that the S351 locus presents a higher phosphorylation level for the control of COAD, UCEC and LUAD (Fig. 4a, b), but not ovarian cancer (P = 6.7e − 1). Moreover, the potential relationship between phosphorylation and the survival prognosis of colorectal cancer was explored. However, we found no significant difference (Fig. S4).

### Immune infiltration analysis

**Fig. 5:**
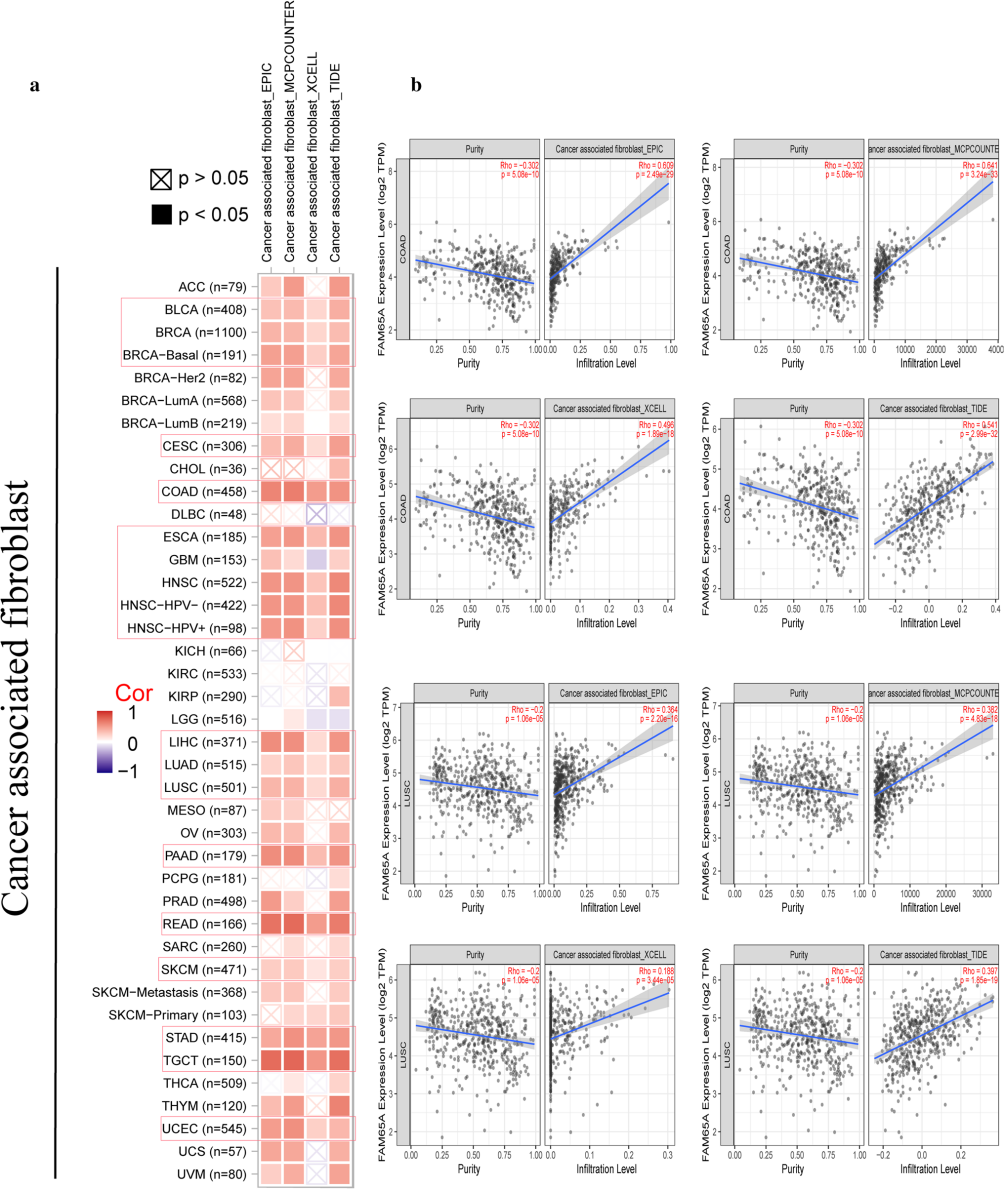
Correlation analysis between FAM65A expression and immune infiltration. **a** The correlation between FAM65A expression and the immune infiltration of CAFs in different tumors is presented based on EPIC, MCPCOUNTED, XCELL and TIDE algorithms. **b** The scatter plot data of COAD and LUSC are presented

In this study, the potential association between *FAM65A* expression and infiltration level of CAFs was investigated based on different algorithms (EPIC, MCPCOUNTED, XCELL and TIDE). We observed that a statistically positive relationship between *FAM65A* expression and the CAFs infiltration for most tumors (Fig. 5a). Additionally, the scatter plots of COAD and LUSC are presented in Fig. 5b.

### Gene enrichment analysis

**Fig. 6:**
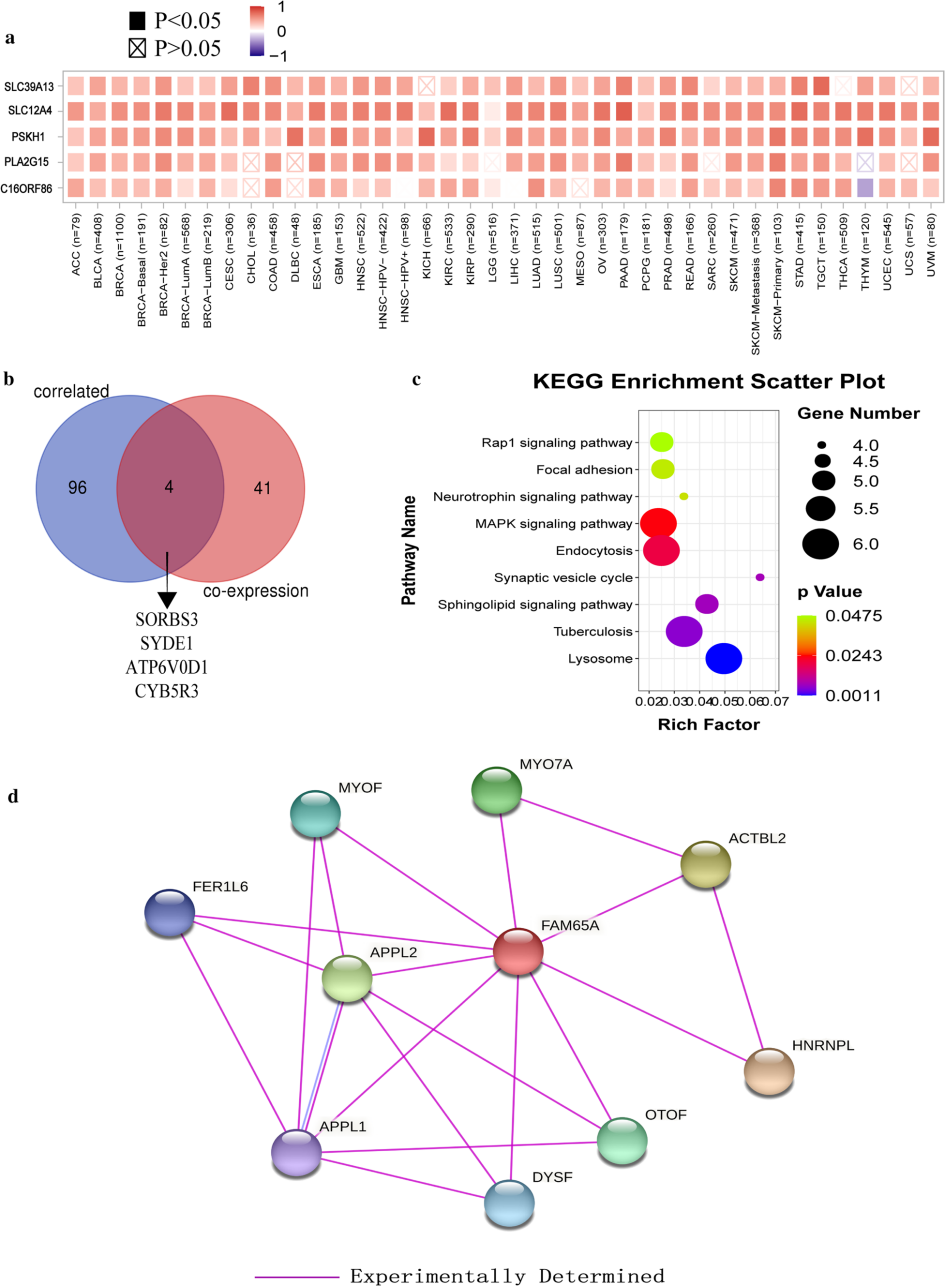
FAM65A-related gene enrichment analysis. **a** The top 100 FAM65A-related genes are obtained through the GEPIA2 tool based on TCGA database and the expression correlation between FAM65A and SLC39A13, SLC12A4, PSKH1, PLA2G15 and C16ORF86 is shown. **b** The intersection between FAM65A correlated and co-expression genes was presented in a Venn diagram, which showed four common members, namely SORBS3, SYDE1, ATP6V0D1 and CYB5R3. **c** KEGG pathway analysis was conducted. **d** The FAM65A-binding proteins confirmed by the experimental methods are presented, using the STRING tool

We tried to screen *FAM65A* correlated and co-expression genes to perform a series of pathway enrichment analysis in order to further explore the *FAM65A* potential mechanisms in human tumors. There is a positive correlation between *FAM65A* and the five genes (SLC39A13, SLC12A4, PSKH1, PLA2G15 and C16ORF86) in most tumors, and the relationship is presented in the heatmap (Fig. 6a). Moreover, the intersection between *FAM65A* correlated and co-expression genes is presented in a Venn diagram, which showed four common members, namely SORBS3, SYDE1, ATP6V0D1 and CYB5R3 (Fig. 6b).

Then, we combined the above genes to conduct KEGG and GO enrichment analyses. KEGG analysis suggested that the combined genes were mainly enriched in “lysosome”, “endocytosis” and “MAPK signalling pathway”, which might participate in the effect of *FAM65A* on tumorigenesis (Fig. 6c). Additionally, GO enrichment analysis indicated most of genes are related to cell physiological activity, such as “regulation of cellular protein localization”, “endosome membrane” and “protein domain specific binding”, and so on (Fig. S5). Finally, the PPI network of FAM65A described by STRING to explore the interacting protein showed 11 nodes, 17 edges, and the PPI enrichment p-value 0.0483 (Fig. 6d).

## Discussion

Pan-cancer analysis is critical and helpful for comparing the similarities and differences between different malignant tumors, which contribute to provide novel insights into tumor biomarkers and cancer prevention. In recent years, many studies have provided a new understanding for whole-genome analysis of human pan-cancer and suggested the close relationship between tumorigenesis and driver genes, mutations, copy number alterations, and tumor purity, which is critical to the diagnosis and treatment of tumors [17–21].

The Rho-family control pathways involved in cellular morphology, which are commonly activated in cancer cell invasion and metastasis [22]. And Rho guanosine triphosphatases (GTPases) have well-recognized roles in regulating various downstream signaling pathways in a wide range of cancers [23]. RHO family interacting cell polarization regulator 1 (RIPOR1) is also known as FAM65A, a novel Rho effector protein, which associates with GTP-bound RHO proteins (RHOA, RHOB, and RHOC) via an N-terminal HR1 domain, plays an important role in RHO-regulated the polarity of migrating cells via Golgi reorientation [8]. In addition, it is reported that FAM65A protein may be a biomarker for cholangiocarcinoma patients [9]. However, the roles of *FAM65A* in human pan-cancer are still unknown. In this study, we explored the correlation of *FAM65A* in different cancer tumorigenesis models by a pan-cancer analysis based on multiple databases. In this study, we first revealed that the *FAM65A* expression is abnormal at mRNA level in most human tumors. This indicates that *FAM65A* may serve as an oncogene in different common cancers. According to KM survival curves, including OS, DFS and DSS, *FAM65A* was confirmed as a potential biomarker of pan-cancer.

Gene mutations play an important role in the pathogenesis of some human tumors [24]. In this study, we observed that the “mutation” was the primary form of DNA alterations of *FAM65A* in all tumors based on the TCGA cohort. It is noteworthy that specific gene mutation is valuable to predict the prognosis of some tumor patients. Phosphorylation is an essential regulatory mechanism in several proteins [25]. The mammalian target of rapamycin (mTOR) is a key regulator of cell growth and proliferation [26]. As early as 2009, the S351 locus phosphorylation of FAM65A was experimentally confirmed in PubMed ID 19276368 [27]. Our findings showed that the expression level of FAM65A total protein phosphorylation at the S351 site was down-regulated in the primary tumors.

A report have shown that cancer-associated fibroblasts (CAFs) play an important role in tumor progression, including carcinogenesis, invasion, metastasis and the chemoresistance of cancer cells [28]. As one of the hallmarks of cancer, development of metastasis accounts for more than 90% cancer-related deaths [29]. It is noteworthy that CAFs secreted exosomes promote metastasis and chemotherapy resistance of colorectal cancer [30, 31]. In this study, we found that *FAM65A* expression was positively associated with CAFs infiltration in colorectal cancer. Therefore, we concluded that the colorectal cancer with high *FAM65A* expression may promote the infiltration level of CAFs, which lead to a poor survival prognosis for the patients.

MAPK cascades are central signalling elements that regulate basic processes including cell proliferation, differentiation and stress responses [32–34]. MAPK signaling is one of the best-defined pathways in cancer biology, and its hyperactivation is responsible for over 40% human cancer cases [35]. In this study, we integrated *FAM65A* correlated and co-expression genes to perform KEGG and GO enrichment analyses, and identified the potential impact of MAPK signalling pathway and MAPK cascade in the pathogenesis of human tumors.

## Conclusions

In summary, our findings contribute to further explore the roles of *FAM65A* in tumorigenesis. Above all, we first indicted that *FAM65A* may be a novel prognostic biomarker of pan-cancer, especially colorectal cancer.

## Supplementary Information

Below is the link to the electronic supplementary material.

Supplementary file 1 (DOCX 1274 KB)

## Data Availability

The raw data are available at GEO: GSE17536.

## Code availability

R 3.5.0, SPSS 23 and Cytoscape 3.8.2.
