# Supplementary material for: FAM65A as a novel prognostic biomarker in human tumors reveal by a pan-cancer analysis
Source: Discov Oncol. 2021 Dec 7;12:60. doi: 10.1007/s12672-021-00456-z (PMC8777545; doi:10.1007/s12672-021-00456-z)
Supplement: Supplementary file 1 — (DOCX 1274 KB) [file 12672_2021_456_MOESM1_ESM.docx]

**SUPPLEMENTARY DATA**

**FAM65A as a novel prognostic biomarker in human tumors reveal by a pan-cancer analysis**

Wenken Liang, Chune Mo, Jianfen Wei, Wei Chen, Weiwei Gong, Jianling Shi, Xianliang Hou, Chunhong Li, Yecheng Deng, Minglin Ou

**Corresponding Author:**

Pro. Minglin Ou

**Address:** Central Laboratory, Guangxi Health Commission Key Laboratory of Glucose and Lipid Metabolism Disorders, The Second Affiliated Hospital of Guilin Medical University, No. 212, Renmin Road, Guilin 541199, China

**E-mail:** minglinou@163.com or minglinou@glmc.edu.cn

**Supplementary Figures**

**
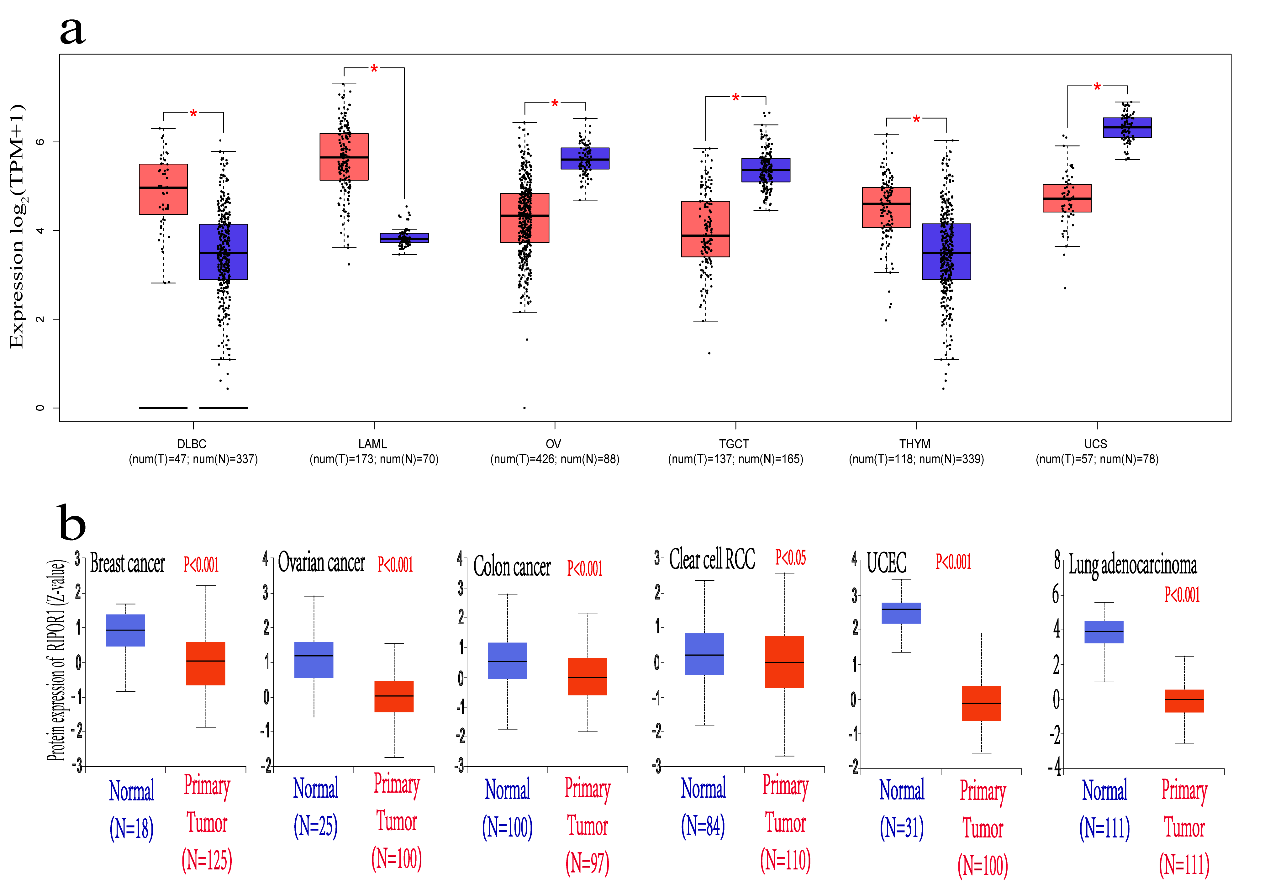
**

**Supplementary Figure S1.** The expression level of FAM65A gene in different tumors. (a) For certain tumors, such as DLBC, LAML, OV, TGCT, THYM and UCS, whose expression information of non-tumor tissues was used as the controls based on GTEx database. (b) The expression levels of the total protein of FAM65A between primary tumor and normal tissue in BLCA, COAD, KICH, OV and THCA are obtained through the UALCAN. * P<0.05, ** P<0.01, *** P<0.001.

**
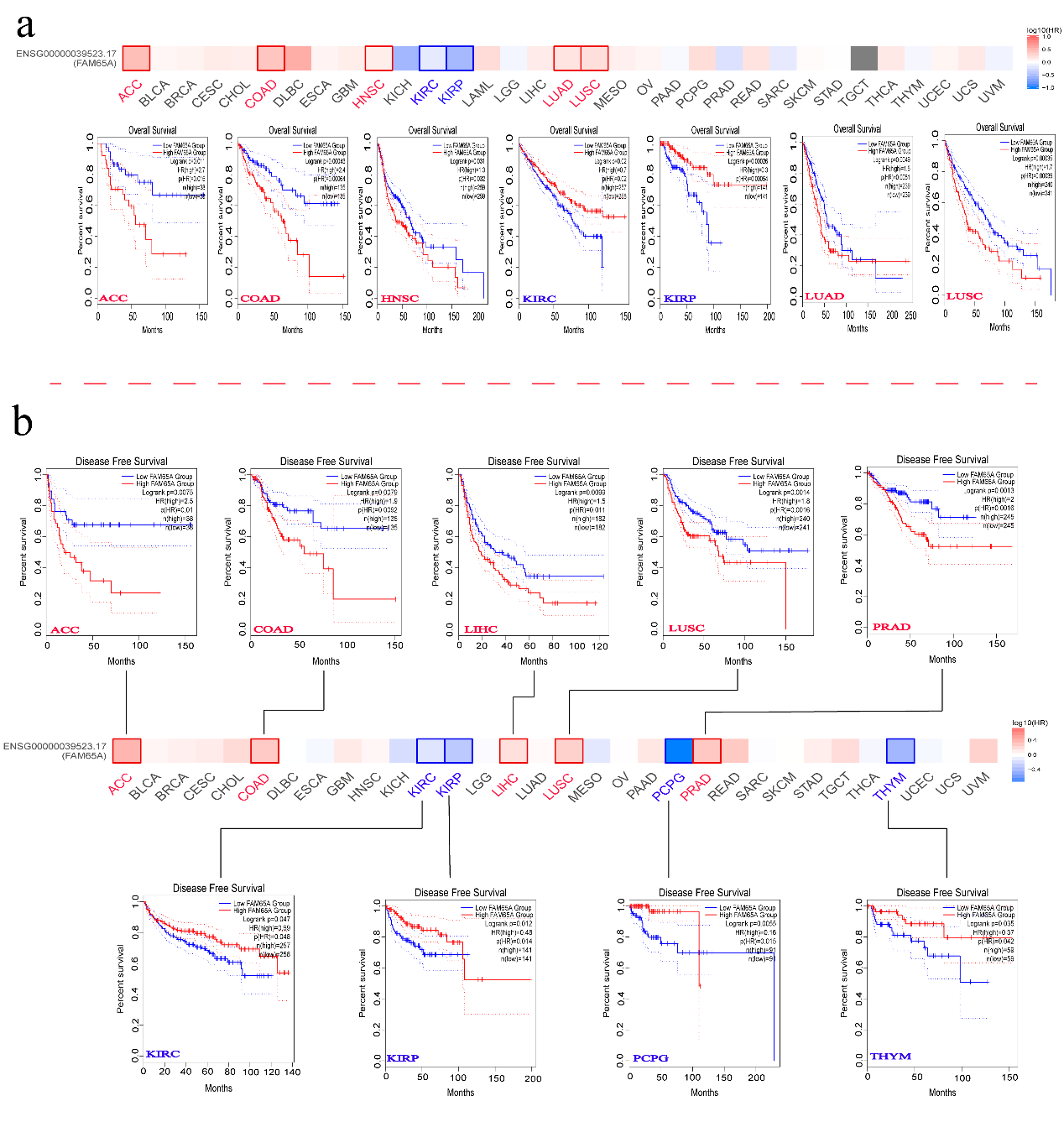
**

**Supplementary Figure S2.** Relationship between FAM65A gene expression and the prognostic values in pan-cancer. (a) Overall Survival; (b) Disease Free Survival

**
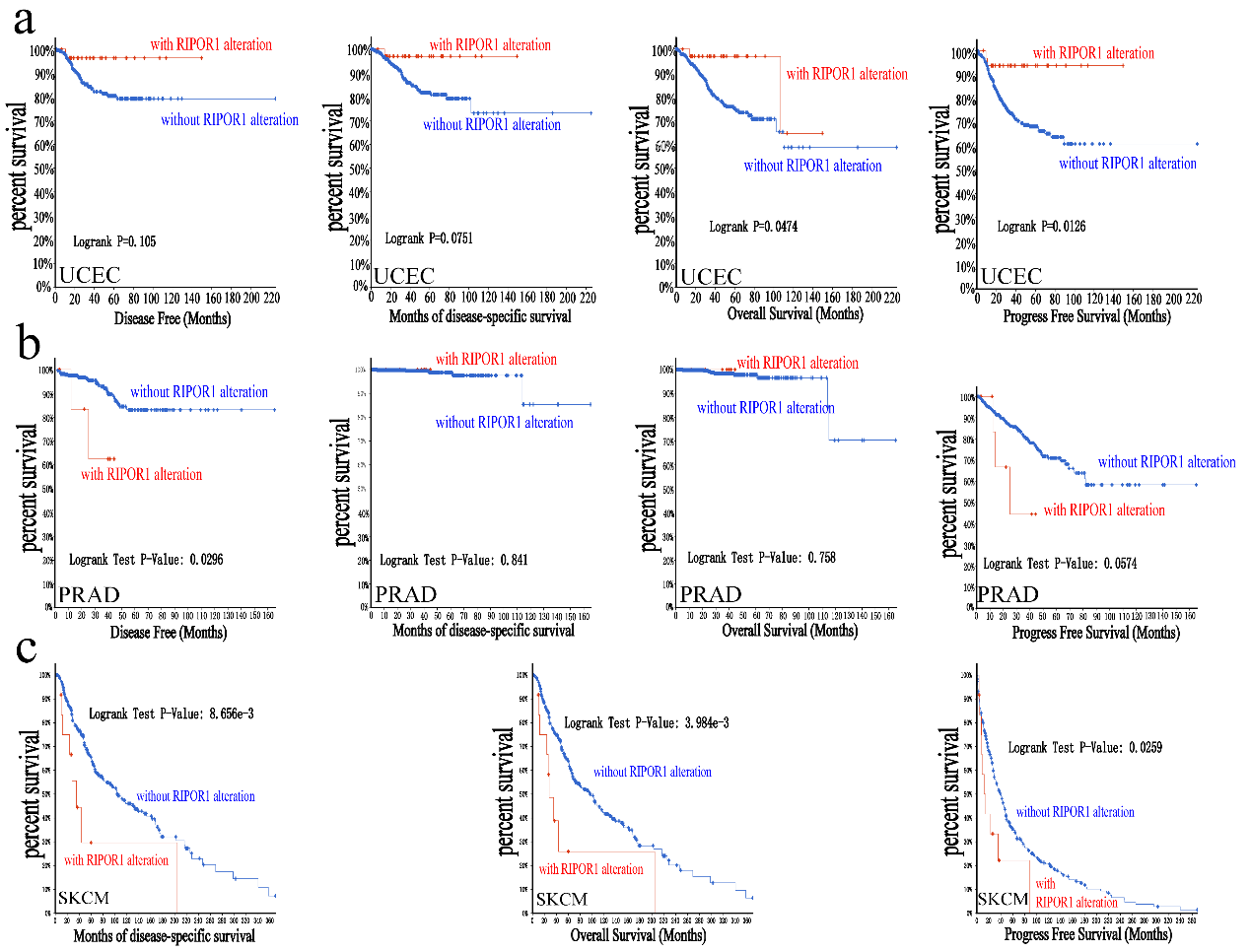
**

**Supplementary Figure S3.** The potential relationship between mutation status and prognostic values of UCEC, PRAD and SKCM.

**
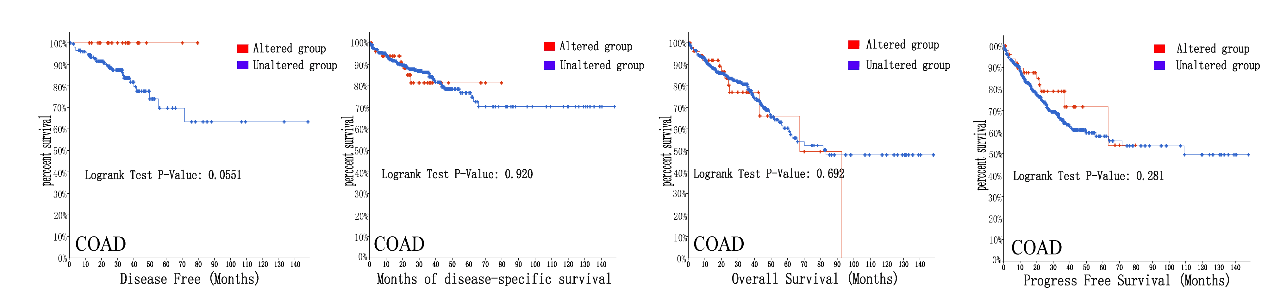
**

**Supplementary Figure S4.** The potential relationship between FAM65A phosphorylation level and the prognostic values of COAD is displayed.


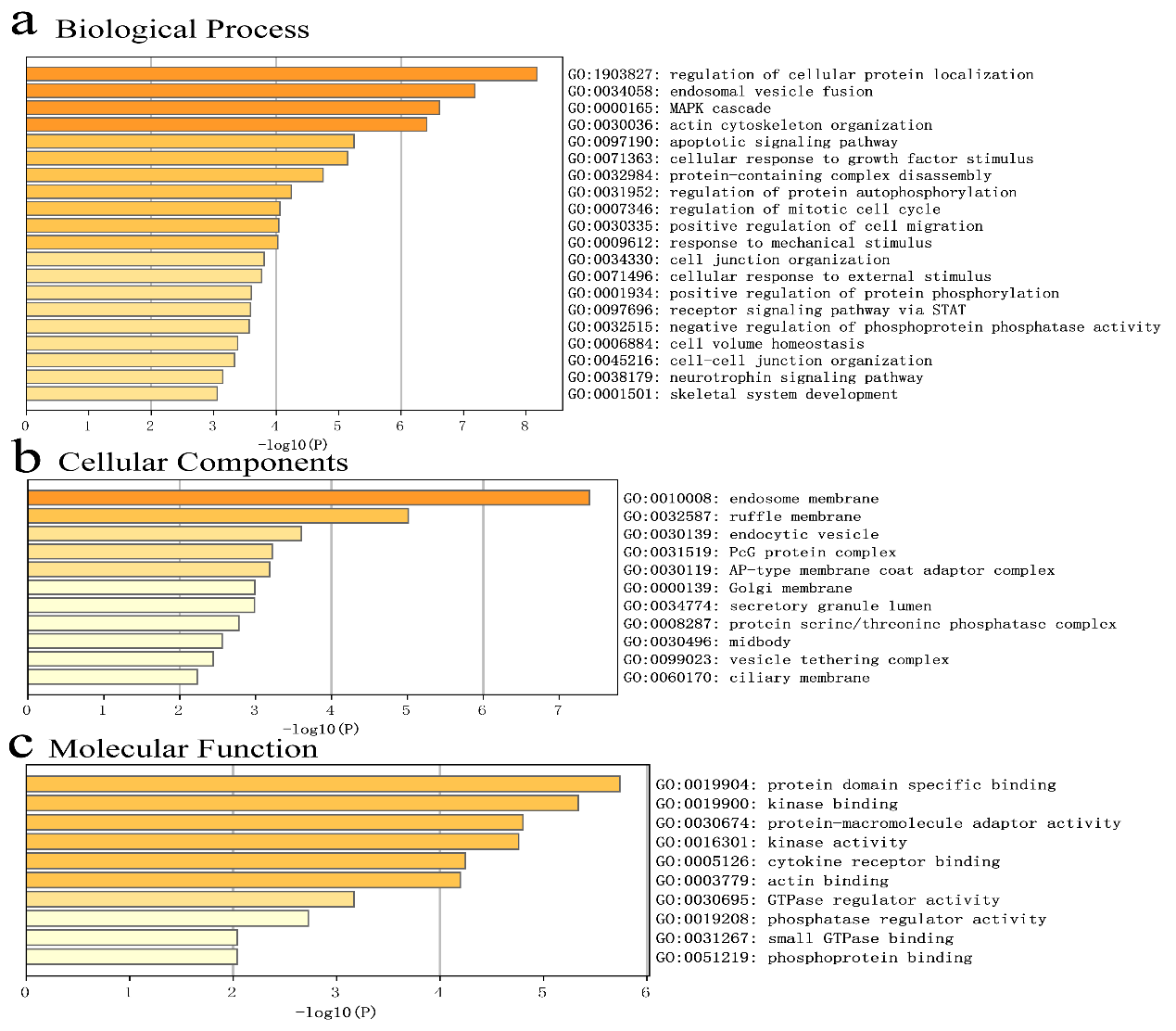


**Supplementary Figure S5.** The GO pathway analysis was conducted. (a) Biological Process; (b) Cellular Components; (c) Molecular Function.
